# Octenyl Succinic Anhydride Modified Pearl Millet Starches: An Approach for Development of Films/Coatings

**DOI:** 10.3390/polym14122478

**Published:** 2022-06-17

**Authors:** Anil Kumar Siroha, Sneh Punia Bangar, Kawaljit Singh Sandhu, Jose Manuel Lorenzo, Monica Trif

**Affiliations:** 1 Department of Food Science and Technology, Chaudhary Devi Lal University, Sirsa 125055, India; siroha01@gmail.com; 2 Department of Food, Nutrition and Packaging Sciences, Clemson University, Clemson, SC 29634, USA; 3 Department of Food Science and Technology, Maharaja Ranjit Singh Punjab Technical University, Bathinda 151001, India; kawsandhu@rediffmail.com; 4 Centro Tecnológico de la Carne de Galicia, Adva. Galicia n° 4, Parque Tecnológico de Galicia, San Cibrao das Viñas, 32900 Ourense, Spain; jmlorenzo@ceteca.net; 5 Área de Tecnología de los Alimentos, Facultad de Ciencias de Ourense, Universidad de Vigo, 32004 Ourense, Spain; 6 CENCIRA Agrofood Research and Innovation Centre, 400650 Cluj-Napoca, Romania; monica_trif@hotmail.com

**Keywords:** starch, OSA, pearl millet, film, coatings, rheology, pasting, digestibility

## Abstract

Pearl millet starches were modified at pH 8.0 using 3.0% octenyl succinic anhydride (OSA), and their pasting, rheological properties, and in vitro digestibility were analyzed. The degree of substitution (D.C.) of OSA-modified starches varied from 0.010 to 0.025. The amylose content decreased after modification, while the reverse was observed for swelling power. After OSA modification, the pasting viscosities (peak, trough, setback (cP)) of the modified starches increased compared to their native counterparts. G′ (storage modulus) and G″ (loss modulus) decreased significantly (*p* < 0.05) compared to their native counterparts during heating. Yield stress (σo), consistency (K), and flow behavior index (n) varied from 9.8 to 87.2 Pa, 30.4 to 91.0 Pa.s., and 0.25 to 0.47, respectively. For starch pastes, steady shear properties showed n < 1, indicating shear-thinning and pseudoplastic behavior. The readily digestible starch (RDS) and slowly digestible starch (SDS) contents decreased, while the resistant starch (R.S.) content increased. After OSA treatment, the solubility power of the starches increased; this property of OSA starches speeds up the biodegradability process for the films, and it helps to maintain a healthy environment.

## 1. Introduction

Pearl millet (*Pennisetum glaucum*) is a widely grown millet worldwide, used in feed and fodder. Pearl millet is a rich source of starch that can be easily isolated from its seed. Native starches have limited shear resistance, thermal resistance, thermal breakdown, and a strong tendency to retrograde, which makes them unsuitable for various commercial food applications [1,2]. As a result, native starches are modified to provide more suitable qualities for food applications. Chemical alterations cause structural changes in starches, improving their physicochemical qualities and allowing them to be used in various industrial applications [3,4]. High viscosity, greater thickening power, minimal gelatinization, and retrogradation are just a few of the benefits of starch succinate [5]. Because of these properties, succinylated starches can be used in a variety of food and non-food applications. In several countries, starch modification with octenyl succinic anhydride (OSA) is currently approved at a dry weight level of 3% for use in food items [6]. OSA starches help to keep an emulsion’s oil–water interface stable. The glucose part of starch binds to the water, and the lipophilic, octenyl part binds to oil, and separation of the oil and water phases is prevented [7]. OSA-modified starches are used in various oil-in-water emulsions for food, pharmaceutical, and industrial products, such as beverages and salad dressings, flavor-encapsulating agents, clouding agents, processing aids, body powders, and lotions [8,9].

A pH of 8.5–9.0, a temperature of 23 °C, and a 5% concentration of OSA were shown to be the best reaction conditions for starch modified with OSA [10]. The use of OSA to esterify starch inhibited the binding of α-amylase, lowering starch digestibility, and this resistant starch could be employed as a functional fiber to treat specific human diseases [11].

Earlier work on the modification of starches by OSA was carried out on corn [12], potato [1], sorghum [13,14], acha [15], pearl millet [16], wheat [17], sweet potato [18], mung bean [19], taro [20], and ginkgo [21]. Starch is the most important polysaccharide for biodegradable film formation because it is cost effective and has better film-forming properties [22,23,24]. There is growing interest in biodegradable films due to consumer preference regarding the dumping of plastic in the environment [25]. Films prepared with OSA starches were observed to have better water barrier characteristics than those made with oxidized and acetylated cross-linked starches [26,27]. The typically hydrophilic starch obtains a hydrophobic ingredient in the form of octenyl groups when treated with OSA, resulting in entire amphiphilic molecules. Emulsification, encapsulation, films and coatings, and gel formation are just a few of the applications of amphiphilic polymers [28].

Pearl millet is an underutilized crop and it has limited industrial applications. It was hypothesized that increasing industrial applications of the underutilized pearl millet crop would boost producer profit. For this, starch was isolated from pearl millet grains and treated with OSA to improve its characteristics. OSA-modified starch has a wide range of industrial applications. However, knowledge is scarce regarding OSA modification of pearl millet starch. The current study was carried out to investigate the physicochemical, rheological, morphological, and in vitro digestibility of OSA-modified pearl millet starch.

## 2. Materials and Methods

Different pearl millet cultivar varieties (HC-10, HHB-67, HHB-223, HHB-226, and W-445) were obtained from Chaudhary Charan Singh Haryana Agricultural University in Hissar (Haryana), India. At the same time, the pearl millet variety GHB-732 was obtained from the Pearl Millet Research Station in Jamnagar (Gujarat, India). The chemicals and reagents [2-Octen-1-ylsuccinic anhydride and Potassium iodide (Sigma-Aldrich, St. Louis, MO, USA), Iodine resublimed (Qualigens, Mumbai, India), Sodium hydroxide (CDH, New Delhi, India), Hydrochloric acid (Rankem, New Delhi, India), Potassium hydroxide (CDH, New Delhi, India), Sodium metabisulphite (CDH, New Delhi, India)] used were of analytical grade.

### 2.1. Starch Isolation

Sandhu and Singh’s approach [29] for isolating starch from pearl millet was followed. Pearl millet grains were steeped for 18–20 h in 0.1 percent sodium metabisulphite solution. The solution was drained and the grains were pulverized in a laboratory grinder when the required time had passed. To eliminate fibrous and proteinious materials, the slurry was sieved through different sieves (0.250, 0.150, 0.100, 0.75 and 0.45 mm). The slurry was centrifuged (Remi, New Delhi, India) for 10 min at 605 g, and the brownish layer was scraped off using a spatula. This technique was repeated until pure starch was obtained.

### 2.2. Preparation of OSA-Modified Starch

The preparation of OSA-modified pearl millet starch was conducted according to a modified version of the method described by Chung et al. [12]. OSA (3 g, 3% based on starch solids) was dissolved in 120 mL of distilled water. In the OSA solution, 100 g/dry basis pearl millet starch was dispersed. The pH of the slurry was adjusted to 8.0 with 1 M HCl or 1 M NaOH, and the reaction was kept at room temperature while stirring for another hour. After that, the pH of the dispersion was adjusted again to 6 with 1 M HCl or 1 M NaOH. Before allowing the samples to air dry, they were washed twice with 450 mL water and once more with 150 mL ethanol.

### 2.3. Degree of Substitutions (D.S.)

The D.S. gives the average amount of hydroxyl groups substituted per glucose unit. Alkali saponification was used to determine it. Whistler and Paschall’s titrimetric approach [30] evaluated the degree of octenyl succinylation in the modified starches. Then, 25 mL of a 0.5 N aqueous NaOH solution was added to the OSA starch suspension (in 50 mL distilled water with 5 g of starch added) and shaken for 24 h. Using phenolphthalein as an indicator, excess alkali was titrated with 0.5 N HCl. Values of the % OSA substitution and D.S. were calculated using Equation (1) and Equation (2), respectively.

(1) $$\%\;\mathrm{OSA}\;\mathrm{Substitution}=\frac{\left(V\;\mathrm{Blank}-V\;\mathrm{Sample}\right)\times 0.1\times \;N\;\times 100}{W}$$

Equation (1): where V blank is the volume of HCl required for blank titration; V sample is the volume of HCl required for sample titration; *W* is the weight of sample taken (g); N is the molarity of HCl solution.

(2) $$\mathrm{DS}=\frac{162\times \%\;\mathrm{OSA}\;\mathrm{Substitution}}{21,000-\left(209\times \%\;\mathrm{Substitute}\right)}$$

Equation (2): where 162 = molecular weight of the glucose unit;

21,000 = 100 × molecular weight of the OS group;

209 = molecular weight of the OS group minus the molecular weight of the hydrogen atom.

### 2.4. Amylose Content (%)

To determine the amylose content of starch, the method described by Williams et al. [31] was used. A total of 10 mL of 0.5 mol/L KOH was thoroughly mixed with 0.020 g starch. The dispersed sample was transferred to a 100 mL volumetric flask and diluted to the desired concentration with distilled water. An aliquot of test starch solution (10 mL) was pipetted into a volumetric flask (50 mL), followed by 5 mL of 0.1 mol/L HCl and 0.5 mL iodine reagent. A spectrophotometer (Thermo Scientific, G1OS UV-Vis, Shanghai, China) was used to measure the absorbance at 625nm after the volume was diluted to 50 mL (Systronics, Ahmadabad, India).

### 2.5. Swelling Power and Solubility (%)

The method described by Leach et al. [32] was used to measure the swelling power and solubility of starches. Starch (1%) was heated in a water bath for 30 min at 90 °C, then cooled to room temperature. The contents were centrifuged for 10 min at 605 g. The swelling power was calculated by weighing the sediments. The supernatants were transferred to pre-weighted dishes, and the contents were dried at 100 °C to determine solubility.

### 2.6. Pasting Properties

The pasting parameter of pearl millet starch was evaluated using a starch cell of a Modular Compact Rheometer (Model-52, Anton Paar, Austria). Starch slurries (1.2 g starch in 13.8 g distilled water) were heated at 50 °C for 1 min, then heated to 95 °C at the rate 6 °C/min. After holding for 2.7 min, the sample was cooled to 50 °C at the same rate and held at 50 °C for 2 min. Each sample was analyzed three times. Peak viscosity (PV), breakdown (BV), setback (SV), final viscosity (FV), and pasting temperature (PT) were all determined using the pasting graph.

### 2.7. Rheological Properties

#### 2.7.1. Dynamic Properties

A Modular Compact Rheometer (Model-52, Anton Paar, Austria) was used to perform small-amplitude oscillatory rheological experiments on different starches utilizing a parallel plate technique (4 cm diameter). Further, 1000 μm was set as the distance between the two spots. The strain and frequency were adjusted to 2% and 10 Hz, respectively, for all measurements. The dynamic rheological properties storage modulus (G′), loss modulus (G″) and loss tangent (tanδ) of starches from various cultivars were examined.

The ram of the rheometer was filled with 15 percent (*w*/*w*) starch suspensions, which were subsequently covered with a thin layer of low-density silicon oil (to minimize evaporation losses). The starch sample were subjected to temperature sweep test and were heated from 45 to 95 °C at the rate of 2 °C/min The starch slurry (10% *w*/*w*) was prepared and manually stirred before being heated in a water bath at 85 °C for 3 min and then stirred again. After allowing the sample to cool to room temperature, it was loaded onto the ram of the rheometer. At 25 °C, frequency sweep tests from 0.1 to 100 rad/s were carried out. At 25 °C, the storage modulus (G′), loss modulus (G″), and loss tangent (tan δ) were calculated.

#### 2.7.2. Steady Shear Measurement

With slight modifications, the method described by Park et al. [8] was used to determine the steady shear properties. The frequency sweep measurement method includes a description of the sample preparation method. The sample (10%) was continuously sheared from 1 to 1000 s^−1^.

The data were fitted to the Herschel–Bulkley model to describe the variation in the rheological properties of samples under steady shear, using the Equation (3):

(3) $$\sigma =\sigma_{o}+K{\left(\dot{\gamma }\right)}^{n},$$

where σ is the shear stress (Pa), σ_o_ is the yield stress, $\dot{\gamma }$ is the shear rate (s^−1^), *K* is the consistency index (Pa.s*^n^*), and *n* is the flow behavior index (dimensionless).

### 2.8. In Vitro Starch Digestibility

The method described by Englyst et al. [33], as modified by Chung et al. [34], was used to determine in vitro starch digestibility. Porcine pancreatic alpha-amylase (Sigma-Aldrich, St. Louis, MO, USA) and amyloglucosidase (Sigma-Aldrich, St. Louis, MO, USA) (3.89 g) were dispersed in water (25.7 mL) and centrifuged for 10 min at 2500 g, yielding 18.7 mL of supernatant. The enzyme solution was supplemented with amyloglucosidase (Sigma-Aldrich, Germany, No. 9913) (1 mL) and deionized water (2 mL). For the digestion analysis, the solution was freshly produced.

In a test tube, guar gum (10 mL, 5 g/L) and sodium acetate (5 mL, 0.5 M) aliquots were added to starch samples (0.5 g, db). After that, each tube was filled with seven glass balls (10 mm in diameter) and 5 mL of enzyme solution, which was then incubated in a water bath (37 °C) with agitation (170 rpm). At intervals, aliquots (0.5 mL) were taken and mixed with 4 mL of 80 percent ethanol. Glucose oxidase and peroxidase assay kits were used to determine the amount of glucose in the combination (No. GAGO-20, Sigma-Aldrich, Germany). The method described by Englyst et al. [33] was used to determine the total starch content of the starch samples.

The starch classification based on its digestibility was: RDS as the starch that was hydrolyzed within 20 min of incubation, R.S. as the starch not hydrolyzed within 120 min, and SDS as the starch digested during the period between 20 and 120 min.

### 2.9. Statistical Analysis

All measurements were recorded in triplicate, and the results were presented as means with standard deviations. Statistical Minitab software was used to perform a one-way analysis of variance (ANOVA) on the data (version 14, Minitab inc., State College, PA, USA).

## 3. Results and Discussions

### 3.1. Degree of Substitution (D.S.)

The D.S. of OSA-modified starches is shown in Table 1. The D.S. of the OSA starches ranged from 0.010 to 0.025%; the highest was observed for cv.HHB-67, whereas cv.W-445 had the lowest value. The degree of insertion of succinyl groups into the starch structure influences the level of succinylation for starches from different cultivars [15]. Awokoya et al. [35] stated that the D.S. is directly influenced by the reactant concentration, reaction duration, pH, and catalyst presence.

### 3.2. Amylose Content (%), Swelling Power (g/g), and Solubility (%)

Amylose content, swelling power and solubility of different OSA-starches differed significantly (*p* < 0.5) (Table 1). The amylose content of OSA starches ranged from 9.3 to 16.1%, cv.HHB-226 had the highest value while cv.GHB-732 had the lowest value. Compared to native starches, the amylose content of the modified starches was lower [36]. Segura-Campos et al. [37] reported that the amylose content of the *P. lunatus* starch decreased after OSA modification, and the introduction of a replacement group with a long hydrophobic chain may improve ramification and, hence, prevent increased absorption of the iodine utilized in the determination technique, resulting in a decrease in amylose content. Many researchers agree with the positive effect of amylose content on the elastic modulus of starch films [25,38,39].

OSA modified starches have swelling powers ranging from 30.7 to 50.0 g/g, cv. HHB-226 had the highest value. OSA-modified starches had a higher swelling power than their native counterparts [36]. Bhosle and Singhal [40] also found that OSA-modified waxy maize and amaranth starches had higher swelling powers than their native counterparts. According to Perez et al. [41], an increase in swelling power could be related to the insertion of the OSA group, reducing intermolecular hydrogen bonds. The solubility of OSA-modified starches ranged from 12.8 to 26.6%, with cv.HC-10 having the highest value, and cv.W-445 having the lowest. After succinylation, the solubility of the starches increased in cv.HC-10, HHB-67, and W-445, but decreased in cv.HHB-223, HHB-226, and GHB-732. Arueya and Oyewale [15] reported that upon succinylation up to 4%, there was an increase in solubilities at all temperatures. Although an exponential decrease in solubility was observed at 7%, solubility began to increase up to 14% addition of succinic anhydride. This may be attributed to structural reorganization, which weakens the starch granules due to the succinylation. The solubility index is dependent on the starch origin and the substitution groups if starch is modified [42]. Higher solubility increases biodegradability and helps in waste management [43].

### 3.3. Pasting Properties

OSA-modified starches had significantly different pasting characteristics (*p* < 0.05) (Table 2). The pasting temperatures for starches ranged from 74.2 to 75.0 °C, with cv.GHB-732 having the highest temperature. The PT determines the minimum temperature necessary to cook the starch. Different OSA starches had PV ranging from 1259 to 2257 cP, with cv.W-445 having the highest PV. Granule swelling is the primary cause of the viscosity increment. Modified starches had FV and SV ranging from 1704 to 3422 cP and 866 to 1477 cP, respectively. The BV of modified starches, which indicates their paste stability, ranged from 57 to 433 cP, cv.W-445 showed the highest value. PV, TV, and SV of the modified starches increased after OSA modification when compared to their native counterpart starches.

According to Shogren et al. [44], the OS group promoted increased water percolation within the granules due to steric hindrance and repulsion, increasing the swelling volume. After the modification, BV and PT were reduced, resulting in an increase in paste stability and a drop in cooking temperature. Bhosale and Singhal [40] reported that PV and SV of starches increased after the OSA modification, while BV and PT decreased. The gelatinization conditions of starch determine the outcome of retrogradation, which affects the film properties, i.e., film crystallinity, drying temperature, relative humidity, and time [45].

### 3.4. Rheological Properties

#### 3.4.1. Dynamic Shear Properties

**Figure 1:**
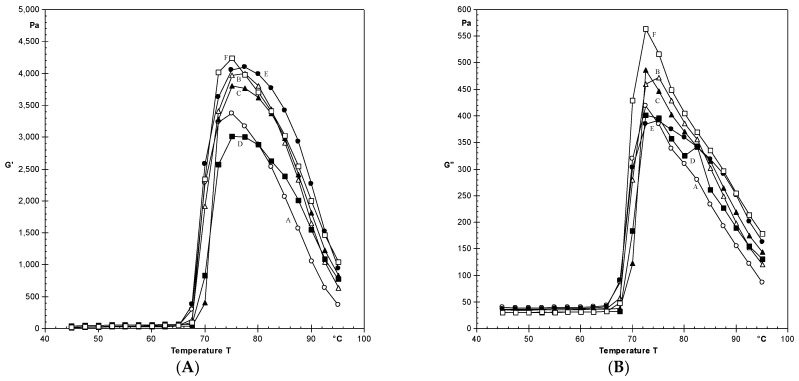
(**A**): Changes in G’ for starches during heating. (**B**): Changes in G″ for starches during heating. Cultivars are denoted by: A: HC-10; B: HHB-67; C: HHB-223; D: HHB-226; E: W-445; F: GHB-732.

Figure 1A,B show variations in the G’, G″, and tan δ of OSA-modified starches of different cultivars following heating in a dynamic rheometer. Starch paste was utilized to determine the rheological parameters, because dry starch has no visco-elastic properties. Flow behavior of starch was determined in this experiment, which was impossible without the use of paste. The temperature at which G′ was maximal (TG′) ranged from 75.0 to 77.5 °C, cv.HHB-67 and cv.W-445 had the highest value. Different starches had peak G′ and G″ values ranging from 3009 to 4234 Pa and 384 to 563 Pa, respectively.

As the temperature increased, G′ and G″ increased, reached a maximum, and then dropped during the heating cycle. During a cycle, the loss factor tan δ (G″/G′) is the ratio of energy lost to the energy retained [46]. OSA-modified starches had loss factors ranging from 0.09 to 0.13.

Breakdown in G′ (difference between peak G′ and value of G′ at 90 °C) ranged from 1466 to 2346 Pa, with cv.HHB-67 and HHB-226 having the highest and lowest values, respectively. G′ and G″ reduced significantly (*p* < 0.05) after OSA modification compared to their native counterparts [36].

**Figure 2:**
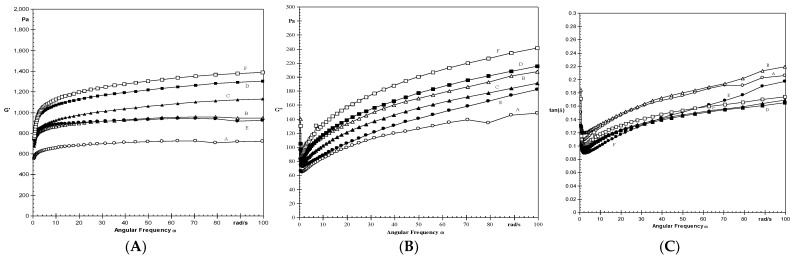
(**A**): Angular frequency dependence of G’ at 25 °C for starches. (**B**): Angular frequency dependence of G” at 25 °C for starches. (**C**): Angular frequency dependence of tan δ at 25 °C for starches. Cultivars are denoted by: (A): HC-10; (B): HHB-67; (C): HHB-223; (D): HHB-226; (E): W-445; (F): GHB-732.

G′, G″, and tan δ change as functions of frequency for OSA-modified starch pastes from different cultivars at 6.28 rad/s and 25 °C, as shown in Figure 2A–C. The peak G′ value of starches ranged from 641 to 1084 Pa, with cv.GHB-732 and cv.HC-10 having the highest and lowest values, respectively. With the increase in frequency, the magnitude of G′ and G″ increased significantly (*p* < 0.05). At all frequencies, the magnitudes of G′ were substantially higher than those of G″, indicating rheological behavior similar to that of weak gels, according to Lee and Yoo [47]. Excluding cv.W-445, where the G″ value was increased, all OSA-modified starches had lower G′ and G″ values than their native counterparts [36]. tan δ values of starches were less than one, indicating that starches are elastic in nature.

#### 3.4.2. Steady Shear Properties

The data of shear stress versus shear rate of OSA-modified starches are shown in Table 3. The experimental data on flow behavior for starch pastes were fitted to the Herschel–Bulkley model, and yield stress (σ_o_), flow behavior index (*n*), and consistency index (*K*) were evaluated. The R^2^ value of starch pastes ranged from 0.999 to 1. The yield stress (σ_o_) value of starch pastes varied from 9.8 to 87.2 Pa; the highest value was observed for cv.HHB-226 and the lowest value was observed for cv.W-445. The σ_o_ value increased for OSA-modified starch pastes from cv.HC-10 and cv.HHB-67, and decreased for the remaining cultivars (HHB-223, HHB-226, W-445 and GHB-732). The *K* value of modified starch pastes varied from 30.4 to 91.0 (Pa.s); the highest and the lowest values were observed for cv.W-445 and cv.HC-10, respectively. The *K* value of modified starch pastes was higher, except for cv.HC-10, when compared to their native counterpart starches [36]. The flow behavior index (*n*) was less than one, describing shear-thinning and pseudoplastic behavior. Sharma et al. [16] reported an increase in σ_o_, *K* and *n* values for OSA-modified starches compared to their native counterpart starches.

### 3.5. In Vitro Digestibility

The readily digestible starch (RDS), slowly digestible starch (SDS), and resistant starch (RS) contents of OSA-modified starches from different cultivars are shown in Table 4.

Octenyl succinylation was known to reduce the enzyme-catalyzed degradation of starch [44,48] and to slow the glycemic response [48]. The RDS content of OSA-modified starches varied from 45.7 to 47.9%, the highest and the lowest values were observed for cv.W-445 and cv.HHB-226, respectively. The SDS and R.S. content of modified starches ranged from 34.2 to 36.2% and 16.5 to 19.9%, respectively; the highest value for cv.HHB.226 and cv.GHB-732, respectively, was observed. RDS is fast and completely digested in the small intestine, and is related to a faster rise in postprandial plasma glucose. On the other hand, SDS digests more slowly in the small intestine and is the most preferred type of dietary starch [49]. After the modification, the RDS and SDS contents were decreased, while the R.S. content was increased compared to native starches [36]. R.S. has potential physiological benefits similar to dietary fiber and unique functional properties [50]. Ai et al. [51] reported that the RDS and SDS contents decreased, and the R.S. content was increased after the OSA modification for normal and high-amylose corn starch.

## 4. Conclusions

This study investigated the structural and rheological properties of OSA-modified pearl millet starch from different cultivars. Swelling power and PV of OSA modified starch ranged between 30.7 to 50.0 g/g and 1259 to 2257 cP, while PT varied from 74.2 to 75.0 °C. Swelling power and PV increased, while PT decreased compared to native counterparts. Understanding rheological characteristics is critical for predicting starch behavior in food products and starch paste movement in pipelines and pumps in the starch industry. The peak G′ value of starches decreased after modification and ranged from 641 to 1084 Pa during the frequency sweep test. The tan δ value of starch pastes was observed less than 1, indicating that the native and modified starch pastes were more elastic than viscous. RDS, SDS and RS content of modified starches varied from 45.7 to 47.9%, 34.2 to 36.2% and 16.5 to 19.9%, respectively. OSA modification adulterates the characteristics of starch, and these properties improve the desirability of starch film. The pasting temperature of OSA-modified starches has decreased, which is beneficial for films and coatings, because this starch requires less energy to gelatinize. Pearl millet, an underutilized crop, could be an excellent substitute for corn starch in starch-based industries.

## Figures and Tables

**Table 1:** Degree of substitution (DS), amylose content, swelling power and solubility of OSA-modified starches.

Cultivars	Degree of Substitution	Amylose Content (%)	Swelling Power (g/g)	Solubility (%)
HC-10	0.023 ± 0.001 ^a^	12.1 ± 0.4 ^c^	41.3 ± 0.5 ^c^	26.6 ± 0.2 ^d^
HHB-67	0.025 ± 0.001 ^a^	13.0 ± 0.3 ^d^	49.2 ± 0.6 ^d^	17.0 ± 0.1 ^c^
HHB-223	0.012 ± 0.002 ^a^	14.3 ± 0.4 ^e^	40.9 ± 0.4 ^c^	14.6 ± 0.1 ^b^
HHB-226	0.013 ± 0.002 ^a^	16.1 ± 0.1 ^f^	50.0 ± 0.5 ^d^	14.4 ± 0.3 ^b^
W-445	0.010 ± 0.002 ^a^	11.0 ± 0.2 ^b^	30.7 ± 0.5 ^a^	12.8 ± 0.1 ^a^
GHB-732	0.011 ± 0.001 ^a^	9.3 ± 0.2 ^a^	36.8 ± 0.5 ^b^	14.0 ± 0.2 ^b^

The values are the averages of three independent determinations. Values in the same column with the same superscript do not differ significantly (*p* < 0.05).

**Table 2:** Pasting properties of OSA-modified starches.

Cultivars	P.V. (cP)	T.V. (cP)	B.V. (cP)	S.V. (cP)	F.V. (cP)	P.T. (°C)
HC-10	1259 ± 11 ^a^	838 ± 9 ^a^	421 ± 5 ^e^	866 ± 7 ^a^	1704 ± 17 ^a^	74.2 ± 0.1 ^a^
HHB-67	1687 ± 13 ^b^	1308 ± 11 ^b^	379 ± 6 ^d^	1293 ± 8 ^d^	2601 ± 22 ^b^	74.4 ± 0.2 ^a^
HHB-223	1925 ± 15 ^c^	1868 ± 13 ^d^	57 ± 3 ^a^	1268 ± 11 ^c^	3190 ± 8 ^d^	74.2 ± 0.1 ^a^
HHB-226	2241 ± 14 ^e^	2000 ± 18 ^f^	241 ± 4 ^c^	1422 ± 10 ^e^	3422 ± 18 ^e^	74.3 ± 0.2 ^a^
W-445	2257 ± 12 ^e^	1824 ± 14 ^c^	433 ± 5 ^e^	1234 ± 13 ^b^	3058 ± 24 ^c^	74.8 ± 0.1 ^b^
GHB-732	2094 ± 16 ^d^	1932 ± 21 ^e^	162 ± 3 ^b^	1477 ± 15 ^f^	3409 ± 22 ^e^	75.0 ± 0.1 ^b^

Values are means of triplicate determinations. Values within the same column followed by the same superscript are not significantly different (*p* < 0.05).

**Table 3:** Steady shear properties of OSA-modified starches fitted with Herschel–Bulkley model.

Cultivars	σ_o_ (Pa)	*K* (Pa.s)	*n*	R^2^
HC-10	61.9 ± 0.5 ^c^	30.4 ± 0.4 ^a^	0.44 ^c^	0.999
HHB-67	43.8 ± 0.6 ^b^	58.0 ± 0.5 ^e^	0.33 ^b^	0.999
HHB-223	59.4 ± 0.5 ^c^	51.3 ± 0.4 ^d^	0.37 ^b^	0.999
HHB-226	87.2 ± 0.7 ^e^	39.2 ± 0.3 ^c^	0.47 ^c^	0.999
W-445	9.8 ± 0.4 ^a^	91.0 ± 0.7 ^f^	0.25 ^a^	0.999
GHB-732	73.7 ± 0.9 ^d^	37.2 ± 0.6 ^b^	0.47 ^c^	1

Values are means of triplicate determinations. Values within the same column followed by the same superscript are not significantly different (*p* < 0.05).

**Table 4:** In vitro digestibility of OSA starches from different cultivars.

Cultivars	RDS (%)	SDS (%)	R.S. (%)
HC-10	47.2 ± 0.4 ^b^	35.6 ± 0.5 ^b^	17.2 ± 0.2 ^a,b^
HHB-67	47.1 ± 0.3 ^b^	36.1 ± 0.3 ^c^	16.8 ± 0.3 ^a^
HHB-223	46.7 ± 0.3 ^a^	35.4 ± 0.2 ^b^	17.9 ± 0.1 ^b^
HHB-226	45.7 ± 0.2 ^a^	36.2 ± 0.2 ^c^	18.1 ± 0.2^b^
W-445	47.9 ± 0.1 ^b^	35.6 ± 0.3 ^b^	16.5 ± 0.1 ^a^
GHB-732	45.9 ± 0.2 ^a^	34.2 ± 0.2 ^a^	19.9 ± 0.2 ^c^

Values are means of triplicate determinations. Values within the same column followed by the same superscript are not significantly different (*p* < 0.05).
